# Optimal gestational weight gain for underweight pregnant women in Japan

**DOI:** 10.1038/s41598-019-54550-y

**Published:** 2019-12-02

**Authors:** Ryosuke Shindo, Mihoko Aoki, Yuriko Yamamoto, Toshihiro Misumi, Etsuko Miyagi, Shigeru Aoki

**Affiliations:** 1 Perinatal Centre for Maternity and Neonates, Yokohama City University Medical Centre, Yokohama, Japan; 2 Department of Biostatistics, Yokohama City University Graduate School of Medicine, Yokohama, Japan; 3 Department of Obstetrics and Gynecology, Yokohama City University School of Medicine, Yokohama, Japan

**Keywords:** Epidemiology, Outcomes research

## Abstract

We aimed to investigate the optimal range of gestational weight gain (GWG) for Japanese underweight (body mass index <18.5 kg/m^2^) women using the Japanese Birth Registry System. The study subjects included underweight women who were divided into groups according to the GWG recommendations of the Ministry of Health, Labour and Welfare (MHLW) (9–12 kg): <9.0 kg, group A; 9–12 kg, group B; and >12 kg, group C. The subjects were then classified according to the recommendations of the Institute of Medicine (IOM) (12.7–18.1 kg): <12.7 kg, group D; 12.7–18.1 kg, group E; and >18.1 kg, group F. In total, 148,135 cases were analysed. The frequencies of small for gestational age, preterm delivery, and caesarean delivery were as follows: 19.3%, 22.7%, and 28.5% for group A; 11.7%, 8.7%, and 22.8% for group B; 8.0%, 4.9%, and 21.5% for group C; 15.0%, 14.7%, and 25.2% for group D; 8.0%, 5.3%, and 21.5% for group E; and 7.0%, 5.5%, and 25.0% for group F, respectively. These results indicated that groups C and E had the best outcomes. Therefore, the IOM guidelines seem more appropriate than the MHLW guidelines. Therefore, the MHLW recommended GWG guidelines require revision.

## Introduction

In the United States and many other developed countries, the increasing prevalence of obesity in pregnant women is regarded as a problem. On the contrary, the problem in Japan is the number of young women who are underweight. According to a survey conducted in 2017, the overall prevalence of underweight women in Japan (body mass index, BMI <18.5 kg/m^2^) was 10.3% and the prevalence of the same in those in their twenties was 21.7%^1^. Pre-pregnancy BMI is related to the birth weight of the child and complications during pregnancy^2^. There is an increased risk of low birth weight^3–8^, foetal growth restriction (FGR)^4,5,9^, threatened preterm delivery, and preterm delivery^3,4,6,10^, and anaemia^3,11^ in pre-pregnant underweight women when compared with those with normal weight before pregnancy. A meta-analysis^12^ reported that women who gain less weight than that recommended by the Institute of Medicine (IOM) during pregnancy have increased risk of preterm delivery and small-for-gestational age (SGA) infants, while women who gain more weight than that recommended have an increased risk of foetal macrosomia, caesarean delivery, and large-for-gestational age (LGA) infants.

Although the recommended gestational weight gain (GWG) for underweight pregnant women is 12.7–18.1 kg^13^, the IOM guidelines were based on women in the United States. Therefore, it is debatable if these guidelines can be applied for Japanese women who have a different physique^14,15^. According to the Ministry of Health, Labour and Welfare (MHLW), the recommended GWG for underweight pregnant women in Japan is 9.0–12.0 kg^2^. While this standard has been set and recommended by expert opinion with respect to the prevention of the development of hypertensive disorder of pregnancy^16^, clear evidence for the same has not been presented. Therefore, we decided to compare the IOM and MHLW guidelines to determine which set of guidelines is appropriate for the Japanese population.

Although the recommended gestational weight gain (GWG) for underweight pregnant women is 12.7–18.1 kg^13^, the IOM guidelines were based on women in the United States. Therefore, it is debatable if these guidelines can be used in Japanese women, who have different physiques^14,15^. While the recommended GWG for underweight pregnant women according to the Ministry of Health, Labour and Welfare (MHLW) in Japan is 9.0–12.0 kg^16^, clear evidence for the same has not been presented.

The objective of the present study was to investigate the appropriateness of MHLW and IOM guidelines regarding the recommended GWG in Japanese underweight pregnant women. This was achieved by conducting a large-scale retrospective study using the Japan Society of Obstetrics and Gynecology (JSOG) Successive Pregnancy Birth Registry System.

## Results

**Figure 1:**
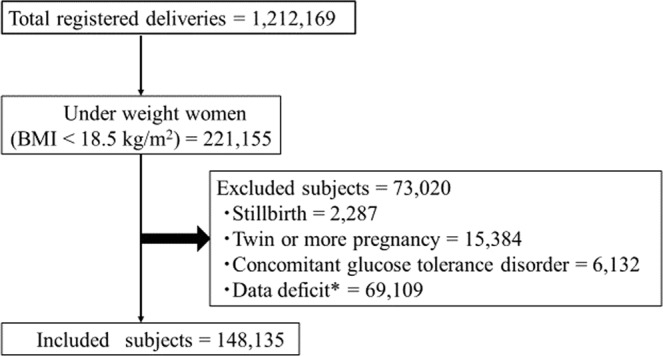
Flow chart for selection of eligible subjects. *Data deficit: Women with missing or apparently incorrect data were excluded. The breakdown of excluded women is summarized as follows (including duplicates): Maternal height; (no data and outside of a range of 120–200 cm) n = 1,850. Maternal pre-pregnancy weight: (no data and outside of a range of 25–100 kg) n = 39,337. Maternal weight at delivery: (no data and outside of a range of 25–100 kg) n = 27,921. Maternal age at delivery: (no data and outside of a range of 10–60 years old) n = 1.

Figure 1 summarizes the study design used. The total number of deliveries between 2007–2015 at the 385 facilities in the JSOG registry system was 1,212,169. Of these, 148,135 cases were analysed in this study. The mean age of the included subjects was 31.0 ± 5.3 years and the mean GWG was 10.3 ± 3.9 kg. The total number of premature deliveries was 18,519 (12.5%) and the total number of Caesarean deliveries was 36,158 (24.4%). There were a total of 19,713 cases (13.2%) of SGA and the mean birthweight was 2796.1 ± 507.1 g. The total number of cases of Hypertensive disorder of Pregnancy (HDP), LGA, and macrosomia were 4,777 (3.2%), 7,822 (5.3%), and 402 (0.3%), respectively.

Table 1 summarizes the background characteristics and outcomes of each group divided according to the GWG recommendations of MHLW. The number of cases (%) in Group A (GWG: <9.0 kg), B (GWG: 9.0–12.0 kg), and C (GWG: >12.0 kg) were 51,171 (34.9%), 56,498 (38.1%), and 40,466 cases (27.3%), respectively. The preterm delivery rates in Groups A, B, and C were 22.7%, 8.7%, and 4.9%, respectively, with Group C having a significantly lower rate (adjusted Odds Ratio (OR): 0.51, 95% CI; 0.48–0.54). The Caesarean delivery rates in Groups A, B, and C were 28.5%, 22.8%, and 21.5%, respectively, and although Group C had the lowest rate, there was no statistically significant difference between Groups B and C (adjusted OR:1.00, 95% CI: 0.98–1.04). The SGA rates in Groups A, B, and C were 19.3%, 11.7%, and 8.0%, with Group C having a significantly lower rate (adjusted OR: 0.64, 95% CI: 0.62–0.68). The low-birthweight (LBW) infant rates in Groups A, B, and C were 34.6%, 16.1%, and 9.2%, respectively, with Group C having a significantly lower rate (adjusted OR: 0.51, 95% CI: 0.49–0.53). The LGA rates in Groups A, B, and C were 2.7%, 4.7%, and 9.4%, respectively, indicating that Group C had a significantly higher rate (adjusted OR: 2.06, 95% CI: 1.96–2.17). However, even in Group C the rate was under 10%. The Apgar score <7 rates in Groups A, B, and C were 2.6%, 0.9%, and 0.8%, indicating that although Group C had the lowest rate, there was no statistically significant difference between Groups B and C (adjusted OR:0.90, 95% CI: 0.79–1.03). The Umbilical arterial (UA) pH < 7.1 rates in Groups A, B, and C were 11.1%, 10.5%, and 10.0%, respectively, indicating that the rate in Group C was significantly lower (adjusted OR: 0.94, 95% CI: 0.90–0.98). However, the HDP rates in Groups A, B, and C were 3.1%, 2.7%, and 4.1%, respectively, indicating that Group B had a significantly lower rate. The mean birthweights in Groups A, B, and C were 2,574 ± 571 g, 2,851 ± 421 g, and 3,001 ± 416 g, respectively, indicating that Group C had the highest birthweight (adjusted regression coefficient (RC): +77, 95% CI: +74–+80).

**Table 1:** Characteristics and Outcomes of groups allotted by the MHLW recommendation.

	A (GWG <9 kg) n = 51,171	“OR * 1 (95 CI * 2) adjusted * 3 OR (95 CI)”	p value	B (9 kg ≦ GWG ≦ 12 kg) n = 56,498	C (GWG> 12 kg) n = 40,466	OR (95% CI) adjusted OR (95% CI)	p value
**Characteristics**							
Maternal age(years), mean ± SD	31.7 ± 5.2			31.2 ± 5.2	29.9 ± 5.5		
Primipara, n (%)	27,773 (54.3%)			31,449 (55.8%)	29,393 (59.2%)		
Maternal height (cm), mean ± SD	158.4 ± 5.5			158.7 ± 5.4	159.3 ± 5.5		
Pre-Pregnancy weight (kg), mean ± SD	44.2 ± 3.6			44.4 ± 3.5	44.5 ± 3.7		
Maternal weight (kg) at delivery, mean ± SD	50.7 ± 4.3			54.9 ± 3.7	59.3 ± 4.6		
Male infants, n (%)	26,007 (50.9%)			28,730 (50.9%)	20,831 (51.5%)		
**Outcome**							
Total preterm delivery (less than 37 weeks)	11,593 (22.7%)	3.06 (2.95–3.17) 3.12 (3.01–3.23)	<0.01 <0.01	4,939 (8.7%)	1,987 (4.9%)	0.54 (0.51–0.57) 0.51 (0.48–0.54)	<0.01 <0.01
Late preterm delivery (34–37 weeks)	6,964 (13.8%	2.18 (2.09–2.27) 2.19 (2.10–2.29)	<0.01 <0.01	3,859 (6.9%)	1,609 (4.0%)	0.56 (0.53–0.60) 0.55 (0.51–0.58)	<0.01 <0.01
Early preterm delivery (less than 34weeks)	4,629 (9.1%)	5.10 (4.77–5.46) 5.27 (4.93–5.64)	<0.01 <0.01	1,080 (1.9%)	378 (0.9%)	0.48 (0.43–0.54) 0.45 (0.40–0.50)	<0.01 <0.01
Caesarean delivery	14,603 (28.5%)	1.35 (1.31–1.39) 1.30 (1.27–1.34)	<0.01 <0.01	12,861 (22.8%)	8,694 (21.5%)	0.93 (0.90–0.96) 1.00 (0.98–1.04)	<0.01 <0.01
Hypertensive disorder of pregnancy	1,588 (3.1%)	1.14 (1.06–1.23) 1.12 (1.04–1.20)	<0.01 <0.01	1,542 (2.7%)	1,647 (4.1%)	1.51 (1.41–1.62) 1.58 (1.47–1.70)	<0.01 <0.01
Low birth weight babies	17,710 (34.6%)	2.77 (2.69–2.85) 2.81 (2.73–2.89)	<0.01 <0.01	9,076 (16.1%)	3,709 (9.2%)	0.53 (0.51–0.55) 0.51 (0.49–0.53)	<0.01 <0.01
Macrosomia	24 (0.05%)	0.28 (0.17–0.43) 0.28 (0.18–0.43)	<0.01 <0.01	95 (0.2%)	283 (0.70%)	4.18 (3.33–5.30) 4.14 (3.29–3.26)	<0.01 <0.01
Small for gestational age	9,872 (19.3%)	1.80 (1.74–1.87) 1.78 (1.73–1.85)	<0.01 <0.01	6,610 (11.7%)	3,231 (8.0%)	0.65 (0.63–0.68) 0.64 (0.62–0.68)	<0.01 <0.01
Large for gestational age	1,383 (2.7%)	0.56 (0.53–0.61) 0.57 (0.54–0.61)	<0.01 <0.01	2,656 (4.7%)	3,783 (9.4%)	2.09 (1.99–2.20) 2.06 (1.96–2.17)	<0.01 <0.01
Apgar score at 5 min < 7	1,319 (2.6%)	2.95 (2.67–3.28) 2.96 (2.67–3.28)	<0.01 <0.01	502 (0.9%)	331 (0.8%)	0.92 (0.80–1.06) 0.90 (0.79–1.03)	0.25 0.13
Umbilical arterial pH< 7.0	4,836 (11.1%)	1.05 (1.01–1.10) 1.05 (1.01–1.10)	0.01 0.01	5,103 (10.5%)	3,468 (10.0%)	0.94 (0.90–0.99) 0.94 (0.90–0.98)	0.01 <0.01
		RC * 4 (95% CI) adjusted RC (95% CI)				RC (95% CI) adjusted RC (95% CI)	
Infant Birth weight (g)	2,574 ± 571	−139 (−141–−136) −138 (−141–−135)	<0.01 <0.01	2,851 ± 421	3,001 ± 416	+75 (+72–+78) +77 (+74–+80)	<0.01 <0.01
Gestational week at delivery (weeks)	37.7 ± 3.0	−0.57 (−0.58–−0.56) −0.57 (−0.58–−0.55)	<0.01 <0.01	38.9 ± 1.8	39.3 ± 1.7	+0.20 (+0.19–+0.24) +0.21 (+0.19–+0.22)	<0.01 <0.01

*1 OR: Odds Ratio for group B.

*2 95% CI: 95% confidence interval.

*3 adjusted for maternal age, height, parity, and infant sex.

*4 RC: Regression coefficient for group B.

Table 2 summarizes the preterm delivery rates for group A′ (expected GWG (e-GWG) <9.0 kg), B′ (9.0 ≦ e-GWG ≦ 12.0 kg), and C′ (e-GWG >12.0 kg). The number of subjects in each group were A′: 41,764 (28.2%), B′: 53,993 (36.5%), and C′: 52,362 (35.4%), respectively. The total preterm delivery rates in Groups A′, B′, and C′ were 16.8%, 10.7%, and 10.9%, respectively, indicating that although Group B′ had the lowest rate, there was no statistically significant difference between Groups B′ and C′ (aOR:0.99, 95% CI: 0.95–1.03). The same results were obtained for late preterm (34–37 weeks) and early preterm (under 34 weeks) deliveries.

**Table 2:** Frequency of preterm delivery: corrected by e-GWG (MHLW).

	A′ (e-GWG<9.0 kg) n = 41,764	OR * 1 (95 CI * 2) adjusted * 3 OR (95 CI)	p value	B′ (9 kg ≦ e-GWG ≦ 12 kg) n = 53,993	C′ (e-GWG>12 kg) n = 52,362	OR * 1 (95 CI * 2) adjusted * 3 OR (95 CI)	p value
Total preterm delivery (less than 37 weeks)	7,005 (16.8%)	1.67 (1.62–1.74) 1.70 (1.64–1.77)	<0.01 <0.01	5,793 (10.7%)	5,721 (10.9%)	1.02 (0.98–1.06) 0.99 (0.95–1.03)	0.3 0.65
Late preterm delivery (34–37 weeks)	4,709 (11.4%)	1.61 (1.54–1.68) 1.62 (1.55–1.69)	<0.01 <0.01	3,967 (7.4%)	3,756 (7.2%)	0.97 (0.93–1.02) 0.96 (0.91–1.00)	0.28 0.06
Early preterm delivery (less than 34weeks)	2,296 (5.5%)	1.66 (1.56–1.77) 1.70 (1.59–1.80)	<0.01 <0.01	1,826 (3.4%)	1,965 (3.8%)	1.11 (1.04–1.19) 1.07 (1.00–1.14)	<0.01 0.06

*1 OR: Odds Ratio for group B.

*2 95% CI: 95% confidence interval.

*3 adjusted for maternal age, height, parity, and infant sex.

Table 3 summarizes the background characteristics and outcomes of each group divided according to the GWG recommendations of IOM. The number of cases in each Groups D (GWG <12.7 kg), E (GWG: 12.7–18.1 kg), and F (GWG >18.1 kg) were 113, 578 (76.7%), 30,888 (20.9%), and 3,669 cases (2.5%), respectively. The preterm delivery rate in Groups D, E, and F were 14.7%, 5.3%, and 5.5%, respectively, indicating that although Group E had the lowest rate, there was no statistically significant difference between Groups E and F (adjusted OR:0.96, 95% CI: 0.82–1.11). The total Caesarean delivery rates in Groups D, E, and F were 25.2%, 21.5%, and 25.0%, respectively, indicating that Group E had a significantly lower rate. The SGA rates in Groups D, E, and F were 15.0%, 8.0%, and 7.0%, respectively, and the LBW rates in the same groups were 24.0%, 9.5%, and 9.0%, respectively, indicating that Group D had significantly higher rates in both. The LGA rates in Groups D, E, and F were 3.9% 9.1%, and 15.3%, indicating that Group F had a significantly higher rate (adjusted OR: 1.81, 95% CI: 1.64–2.00) and that Group D had a significantly lower rate (adjusted OR: 0.42, 95% CI: 0.40–0.44). Nevertheless, the rate in Group E was under 10%. The Apgar score <7 rates in Groups D, E, and F were 1.7%, 0.9%, and 1.1%, respectively, indicating that Group D had a significantly higher rate. Similarly, the umbilical artery pH < 7.1 rates in Groups D, E, and F were 10.8%, 10.0%, and 9.6%, respectively, indicating that Group D had a significantly higher rate. The HDP rates in Groups D, E, and F were 2.9%, 4.1%, and 6.8%, respectively, indicating that Group D had a significantly lower rate and that Group F had a significantly higher rate (aOR:0.67, 95% CI:0.62–0.71 and aOR:1.78, 95% CI:1.54–2.06, respectively). The mean birthweights in Groups D, E, and F were 2,733 ± 512 g, 2,994 ± 422 g, and 3,082 ± 479 g, respectively, indicating that Group F had a significantly higher mean birthweight (adjusted RC: +51, 95% CI: +43–+ 59).

**Table 3:** Characteristics and Outcomes of groups allotted by the IOM recommendation.

	D (GWG< 12.7 kg) n = 113,578	“OR * 1 (95 CI * 2) adjusted * 3 OR (95 CI)”	p value	E (12.7 kg ≦ GWG ≦ 18.1 kg) n = 30,888	F (GWG >18.1 kg) n = 3,669	OR (95 CI%) adjusted OR (95% CI)	p value
**Characteristics**							
Maternal age(years), mean ± SD	31.4 ± 5.2			29.9 ± 5.5	28.9 ± 5.9		
Primipara, n (%)	62,621 (55.2%)			18,192 (59.0%)	2,348 (64.1%)		
Maternal height (cm), mean ± SD	158.6 ± 5.5			159.3 ± 5.5	159.9 ± 5.6		
Pre-Pregnancy weight (kg), mean ± SD	44.2 ± 5.5			44.6 ± 3.7	44.2 ± 4.3		
Maternal weight (kg) at delivery, mean ± SD	53.1 ± 4.5			59.0 ± 4.0	65.4 ± 5.3		
Male infants, n (%)	57,718 (50.9%)			15,904 (51.5%)	1,946 (53.1%)		
**Outcome**							
Total preterm delivery (less than 37 weeks)	16,677 (14.7%)	3.07 (2.91–3.24) 3.20 (3.03–3.37)	<0.01 <0.01	1,639 (5.3%)	203 (5.5%)	1.04 (0.90–1.21) 0.96 (0.82–1.11)	0.6 0.01
Late preterm delivery(34–37weeks)	11,001 (9.8%)	2.49 (2.35–2.64) 2.55 (2.40–2.70)	<0.01 <0.01	1,283 (4.2%)	148 (4.0%)	0.97 (0.81–1.15) 0.91 (0.76–1.08)	0.74 0.29
Early preterm delivery (less than 34weeks)	5,676 (5.0%)	4.51 (4.06–5.03) 4.80 (4.31–5.36)	<0.01 <0.01	356 (1.2%)	55 (1.5%)	1.30 (0.97–1.72) 1.15 (0.85–1.54)	0.07 0.34
Caesarean delivery	28,595 (25.2%)	1.23 (1.19–1.26) 1.11 (1.08–1.14)	<0.01 <0.01	6,647 (21.5%)	916 (25.0%)	1.21 (1.20–1.31) 1.30 (1.21–1.42)	<0.01 0.67
Hypertensive disorder of pregnancy	3,279 (2.9%)	0.70 (0.66–0.75) 0.67 (0.62–0.71)	<0.01 <0.01	1,250 (4.1%)	248 (6.8%)	1.72 (1.49–1.98) 1.78 (1.54–2.06)	<0.01 <0.01
Low birth weight babies	27,246 (24.0%)	3.02 (2.90–3.15) 3.09 (2.97–3.22)	<0.01 <0.01	2,920 (9.5%)	329 (9.0%)	0.94 (0.84–1.06) 0.86 (0.76–0.97)	0.34 0.86
Macrosomia	144 (0.1%)	0.20 (0.17–0.26) 0.21 (0.17–0.26)	<0.01 <0.01	1,87 (0.6%)	71 (1.9%)	3.24 (2.45–4.25) 3.37 (2.53–4.43)	<0.01 <0.01
Small for gestational age	16,999 (15.0%)	2.03 (1.95–2.13) 2.02 (1.93–2.12)	<0.01 <0.01	2,457 (8.0%)	257 (7.0%)	0.87 (0.76–0.98) 0.81 (0.71–0.93)	0.03 <0.01
Large for gestational age	4,456 (3.9%)	0.41 (0.39–0.43) 0.42 (0.40–0.44)	<0.01 <0.01	2,806 (9.1%)	560 (15.3%)	1.80 (1.63–1.99) 1.81 (1.64–2.00)	<0.01 <0.01
Apgar score at 5 min < 7	1,850 (1.7%)	1.92 (1.70–2.20) 1.95 (1.72–2.24)	<0.01 <0.01	263 (0.9%)	39 (1.1%)	1.25 (0.88–1.74) 1.19 (0.84–1.08)	0.2 0.32
Umbilical arterial pH< 7.0	10,473 (10.8%)	1.09 (1.04–1.14) 1.09 (1.04–1.14)	0.01 0.01	2,637 (10.0%)	297 (9.6%)	0.96 (0.85–1.09) 0.96 (0.84–1.08)	0.57 0.52
		RC*4 (95% CI) adjusted RC (95% CI)				RC (95% CI) adjusted RC (95% CI)	
Infant Birth weight (g)	2,733 ± 512	−131 (−134–−128) −138 (−134–−128)	<0.01 <0.01	2,994 ± 422	3,082 ± 479	+44 (+35–+52) +51 (+43–+59)	<0.01 <0.01
Gestational week at delivery (weeks)	38.4 ± 2.5	−0.43 (−0.44–−0.41) −0.43 (−0.44–−0.41)	<0.01 <0.01	39.2 ± 1.6	39.3 ± 3.1	+0.02 (−0.02–+0.06) +0.03 (+0.01–+0.07)	0.36 <0.01

*1 OR: Odds Ratio for group B.

*2 95% CI: 95% confidence interval.

*3 adjusted for maternal age, height, parity, and infant sex.

*4 RC: Regression coefficient for group B.

Table 4 summarizes the preterm delivery rates for groups D′ (e-GWG < 12.7 kg), E′ (12.7 ≦ e-GWG ≦ 18.1 kg), and F′ (e-GWG >18.1 kg). The number of cases in Groups D′, E′, and F′ were 105,703 (71.36%), 37,166 (25.09%), and 5,250 (3.54%), respectively. The preterm delivery rates in Groups D′, E′, and F′ were 13.1%, 10.4%, and 14.6%, respectively, indicating that Group E′ had a significantly lower rate(adjusted OR: 1.34, 95% CI: 1.29–1.39 and adjusted OR: 1.40, 95% CI: 1.29–1.53, respectively). The results for late preterm (34–37 weeks) and early preterm (under 34 weeks) deliveries were the same.

**Table 4:** Frequency of preterm delivery: corrected by e-GWG (IOM).

	D′ (e-GWG <12.7 kg) n = 105,703	OR * 1 (95% CI * 2) adjusted * 3 OR (95 CI)	p value	E′ (12.7 kg ≦ e-GWG≦ 18.1 kg) n = 37,166	F' (e-GWG >18.1 kg) n = 5,250	OR * 1 (95 CI * 2) adjusted * 3 OR (95% CI)	p value
Total preterm delivery (less than 37 weeks)	13,885 (13.1%)	1.34 (1.25–1.35) 1.34 (1.29–1.39)	<0.01 <0.01	38,67 (10.4%)	767 (14.6%)	1.47 (1.35–1.60) 1.40 (1.29–1.53)	<0.01 <0.01
Late preterm delivery (34–37weeks)	9,422 (9.0%)	1.32 (1.26–1.38) 1.34 (1.28–1.40)	<0.01 <0.01	2,570 (7.0%)	440 (8.5%)	1.24 (1.11–1.38) 1.18 (1.06–1.31)	<0.01 <0.01
Early preterm delivery (less than 34weeks)	4,463 (4.2%)	1.22 (1.15–1.30) 1.27 (1.19–1.36)	<0.01 <0.01	1,297 (3.5%)	327 (6.2%)	1.72 (1.53–1.97) 1.74 (1.53–1.97)	<0.01 <0.01

*1 OR: Odds Ratio for group B.

*2 95% CI: 95% confidence interval.

*3 adjusted for maternal age, height, parity, and infant sex.

## Discussion

Japanese underweight pregnant women had preferable pregnancy outcomes when they had greater GWG than that recommended by the MHLW (12.0 kg). Moreover, they had good pregnancy outcomes when they had a GWG similar to that recommended by the IOM (12.7–18.1 kg). Therefore, we recommend that Japanese underweight pregnant women should have a GWG in the range of 12.0–18.1 kg.

Japanese underweight pregnant women who achieved excess GWG (>12 kg: group C) had significantly lower frequencies of preterm deliveries, SGA and LBW than women who achieved a GWG that was recommended by the MHLW (9–12 kg: group B). However, there was no significant difference between the recommended GWG group and the excess GWG group when the frequency of preterm birth was corrected by e-GWG. In contrast, the frequency of LGA increased (C 9.4% vs B 4.7%). The fact that even with excess GWG (>12 kg), the LGA frequency remained <10%, suggests the possibility that the GWG recommended by the MHLW is insufficient in ensuring appropriate foetal growth. Although there was a slight increase in the frequency of HDP with excess GWG, the frequencies of caesarean deliveries and umbilical-artery pH < 7.1 did not increase. In fact, these frequencies were the lowest in women with excess GWG. These data suggest that excess GWG (>12 kg) may be the optimal weight gain for underweight Japanese pregnant women, and not that recommended by the MHLW (9–12 kg). A retrospective study in Japanese underweight pregnant women found that women with BMIs between 17.0 and 18.4 kg/m^2^ had good pregnancy outcomes when their GWG was 12.2 kg (10.8–13.6 kg) at 40 weeks, and that the GWG recommended by the MHLW (9–12 kg) was insufficient^17^. A retrospective study of 1,559 underweight (BMI <18.5 kg/m^2^) Japanese pregnant women demonstrated that higher GWG than that recommended by the MHLW (>12.0 kg) decreased the incidence of SGA (OR:0.68, 95% CI: 0.43–1.06)^14^. The results of the present study support the findings of these prior studies. With less GWG (<9 kg), the frequencies of preterm deliveries, caesarean deliveries, Apgar scores <7, and umbilical-artery pH < 7.1 were higher, and the delivery outcomes were poorer than those with recommended GWGs (9–12 kg). These results are consistent with the results of previous studies^2,12,17–21^ that reported that insufficient weight gain during pregnancy increases the risk of SGA and preterm delivery. Investigation of the relationship between less GWG and preterm delivery indicated that even with e-GWG, which accounts for pregnancy duration, there was a significant increase in the frequency of preterm deliveries; this further indicates that less GWG (<9 kg) is a dependent risk factor for preterm delivery.

The GWG recommended by the IOM (12.7–18.1 kg) resulted in optimal pregnancy outcomes even in Japanese underweight women. With less GWG (<12.7 kg: group D), there was a higher preterm delivery rate than that observed with the recommended GWG (12.7–18.1 kg: group E). Even when the results were adjusted using e-GWG, there was a statistically significant difference. Additionally, with less GWG (<12.7 kg), there was a significantly higher frequency of SGA than that observed when GWG was within the recommended range. Conversely, although the LGA frequency decreased (D 3.9% vs. E 9.1%), the LGA frequency within the recommended GWG (12.7–18.1 kg) group remained under 10%. This suggests that the recommended GWG range of 12.7–18.1 kg is appropriate for acceptable foetal growth. Although the HDP frequency increased when the GWG was within the recommended range of 12.7–18.1 kg in comparison to that observed when GWG was less, the frequencies for caesarean deliveries, Apgar scores <7.0, and UA pH < 7.1 were significantly lower; thus, overall, the delivery outcomes in women with GWG within the recommended range (12.7–18.1 kg) were good. With excess GWG (>18.1 kg: group F), the rates of caesarean deliveries, HDP, macrosomia, and LGA were significantly higher than those observed with the GWG within the recommended range of 12.7–18.1 kg, and the delivery outcomes were poor. This indicates that in Japanese underweight women, a GWG of 18.1 kg or more is not appropriate.

Several studies have investigated whether the IOM guidelines are appropriate for Japanese underweight pregnant women. The results of the present study were consistent with the findings of a past study, which divided 17,724 underweight pregnant women into groups of Less, Recommended, and Excess GWG based on the IOM guidelines, and reported that the IOM-recommended GWG group was the most appropriate for good delivery outcomes^2^. In a study in which 515 Japanese underweight pregnant women were divided into two groups based on the MHLW guidelines and the IOM guidelines, Suzuki reported that underweight pregnant women in the IOM-guideline group had higher incidence of GDM but lower incidences of preterm delivery and LBW, indicating that the IOM guidelines were more optimal than the MHLW guidelines^18^. These findings are consistent with those of the present study.

This study had several limitations. First, the facilities registered with the database utilized consisted only of tertiary medical facilities, which indicates the possibility of selection bias because a large number of high-risk pregnancies were included. Second, because the data stretched over a nine-year period, chronological changes in the background circumstances may have occurred. Third, we did not examine the long-term outcomes of either the pregnant women or their infants who were included in this study. Nevertheless, as this was a large-scale retrospective study of 149,135 cases, the fact that we were able to evaluate the preterm delivery rates that were adjusted for gestational week is a major merit of this study.

In conclusion, the results of this study suggest that the optimal GWG for Japanese underweight women is closer to the IOM guidelines than it is to the MHLW guidelines. The determination of optimal weight gain will differ based on the priorities, such as prevention of SGA, LBW infants, and preterm delivery rates—all of which are common in underweight pregnant women. If all are selected as priorities, then the IOM guidelines seem more appropriate than the MHLW guidelines. Therefore, the MHLW recommended GWG guidelines require revision.

## Methods

### Ethical approval and informed consent

This study was approved by the Ethics Committee of Yokohama City University Medical Centre. Because this study used anonymized databases in which the opt-out consent method was adopted, obtaining informed consent from individuals was not required. Adopting the opt-out form instead of individual informed consent has been approved by the Ethics Committee.

### Study design

In this retrospective study, we used the perinatal registration database of the JSOG, which is known as the JSOG Successive Pregnancy Birth Registry System. The JSOG Successive Pregnancy Birth Registry System was initiated by JSOG in 2001. It contains anonymized data on deliveries occurring after a minimum of 22 gestational weeks from participating facilities throughout Japan. In 2007, a total of 117 facilities were registered with the system and an approximate total of 60,000 deliveries (5.8% of all deliveries in Japan) were included in the system. Each year an increasing number of facilities are registered with the system. In 2015, the registration of 385 facilities resulted in an increase in the number of deliveries included in the system to approximately 240,000, which represented 23.8% of all deliveries in Japan that year. The present study utilized data collected by this system between 2007 and 2015.

### Participants

#### Inclusion criteria

Pregnant women with singleton pregnancies and pre-pregnancy BMI <18.5 kg/m^2^ were selected from the total number of pregnant women registered in the system and included in this study.

#### Exclusion criteria

Cases of stillbirth, pre-pregnancy diabetes mellitus (DM), gestational diabetes mellitus (GDM), overt diabetes mellitus (overt DM), cases with missing data, and cases of clear outlier data were excluded. Outlier data were defined as follows:

- Maternal height: Outside the range of 120–200 cm.
- Maternal weight: Outside the range of 25–100 kg.
- Maternal age at delivery: Outside the range of 10–60 years.

#### Group allotment

The subjects were allotted to groups based on the gestational weight gain (GWG) recommended by the MHLW guidelines and IOM guidelines, and the groups were compared.

- Investigation 1: Classification based on GWG recommended by MHLW
  The MHLW guidelines recommend GWG of 9–12 kg. Therefore, the groups included: Group A: GWG <9 kg, Group B: 9 ≦ GWG ≦ 12 kg, Group C: GWG >12 kg. The pregnancy outcomes of these three groups were compared.
- Investigation 2: Classification based on GWG recommended by IOM

The IOM guidelines recommends GWG of 12.7–18.1 kg. Therefore, the groups included, Group D: GWG <12.7 kg, Group E: 12.7 ≦ GWG ≦ 18.1 kg, Group F: GWG >18.1 kg. The pregnancy outcomes of these three groups were compared.

Lower GWG is an inevitable consequence of shorter gestational length in preterm deliveries. However, without adjusting the data, it is difficult to accurately assess the relationship between gestational weight gain and preterm delivery. In the present study, we utilized the following method of data adjustment, which was reported by Morisaki *et al*.^17^. Based on the data of pre-pregnancy weight, gestational weeks, and weight at delivery of 1,283 Japanese pregnant women whose detailed weight data during pregnancy were recorded, we used a formula to estimate the GWG (expected-GWG: e-GWG) had the pregnancy continued until delivery at 40 weeks. Using this method, we calculated e-GWG with the assumption that all pregnant women delivered at 40 weeks.

We investigated the relationship between e-GWG and preterm delivery by comparing the following groups:

Investigation 3 Group A′: e-GWG <9 kg, Group B′: 9 ≦ e-GWG ≦ 12 kg, Group C′: e-GWG > 12 kg

Investigation 4 Group D′: e-GWG <12.7 kg, Group E′: 12.7 ≦ e-GWG ≦ 18.1 kg, Group F′: e-GWG >18.1 kg

### Maternal and neonatal characteristics

The maternal and neonatal characteristics included pre-pregnancy weight (kg), height (cm), weight at delivery (kg), maternal age at delivery (years), and sex of the child.

### Pregnancy outcomes

We compared the following pregnancy outcomes in all groups: Gestational weeks at delivery, total premature deliveries (under 37 weeks), late preterm deliveries (34-under 37 weeks), early preterm deliveries (under 34 weeks), Caesarean deliveries (total), emergency Caesarean deliveries, hypertensive disorders of pregnancy (HDP), SGA, LGA, and low birthweight infants (<2500 g), macrosomia (≥4000 g), weight at birth, umbilical artery (UA) pH < 7.1, and Apgar Score <7.0 (at 5 mins).

SGA was defined as a neonatal birth weight below the 10^th^ percentile of the standard weight for each gestational week based on the sex of the infant and the corresponding birth order. LGA was defined as a neonatal birth weight equal to or above the 90^th^ percentile of the same criterion. HDP was defined as hypertension during pregnancy (systolic blood pressure [BP] ≧140 mmHg or/and diastolic BP ≧90 mmHg). HDP included preeclampsia, gestational hypertension, superimposed preeclampsia, chronic hypertension, and/or renal diseases.

### Statistical analysis

JMP® Pro 12.2.0 (SAS Institute Inc.) was used for the statistical analyses. The maternal characteristics were expressed as means ± standard deviation (SD) or frequencies (%). The outcomes between the groups were compared using logistic regression analysis and multiple regression analysis was used for the continuous variables. We calculated the odds ratio (OR) or regression coefficient (RC) and 95% confidence intervals (CI) with reference to the group used as the controls. We performed multivariate analysis in order to adjust for the confounding factors. We calculated the adjusted odds ratio (adjusted OR) or the adjusted regression coefficient (adjusted RC) in order to adjust for the following confounding factors: maternal age, height, pre-pregnancy weight, parity, and sex of the infant. The controls were the weights recommended by the MHLW and IOM guidelines (group B in investigation 1, E in investigation 2, B′ in investigation 3, and E′ in investigation 4).

## Data Availability

The data that support the findings of this study are available from the Japan Society of Obstetrics and Gynecology (JSOG) but restrictions apply to the availability of these data, which were used under license for the current study, and so are not publicly available. Data are however available from the authors upon reasonable request and with permission of the JSOG.
